# Supplementary material for: Patterns of Intron Gain and Loss in Fungi
Source: PLoS Biol. 2004 Nov 30;2(12):e422. doi: 10.1371/journal.pbio.0020422 (PMC532390; doi:10.1371/journal.pbio.0020422)
Supplement: Table S1 — Also available at http://genes.mit.edu/NielsenEtAl/. (4.3 MB ZIP). [file pbio.0020422.st001.zip › NielsenEtAl/html/1252.html]

AN7744.1.NCU00127.1.MG04836.1.FG07271.1


```
 CLUSTAL W (1.82) Multiple Sequence Alignments - Introns Inserted


Sequence 1: AN7744.1	425 aa
Sequence 2: FG07271.1	487 aa
Sequence 3: NCU00127.1	591 aa
Sequence 4: MG04836.1	487 aa
Alignment Length: 619 aa
Number Identitical Residues: 66 aa
Alignment Score (without introns) 3920


MG04836.1 	------MPTIASTSTAPPPPLLSAAPPGPGLRRSANGNGNGNGNGHGTNGGPHRRRSNIG
NCU00127.1	------MATVTSTETEDTPAMSPSTLDSRPMRRAAT-------IGEGSQPAQLRRR----
FG07271.1 	MADENTTSNSSGRPAAGVSSGFNAPPSGQGPRRAMT-------VSVAEEAASASRRQYPA
AN7744.1  	------MSNSPS------PSPLKPTLRHRGLARAAT-------VAEGPQPQIPPQRRN--
          	       .. ..      ..   ..       *: .        . . :     :*    

MG04836.1 	TTGEPSAGNGNGFDHHRRRYSNVSSDPDTFDIRSLGPDFP-PDTFDKPMRQQLLNWQSVP
NCU00127.1	-LVNSQSTLGTSFGEFRRRSSTFSDYSLDAARRNLHHEILNPAASFDSDEHGASESSLMP
FG07271.1 	SPSFDSIP--------PRRSSNFSEYSLNEARDFLNPQPRDPGNADSSLAGESSSLPSLS
AN7744.1  	STFSDSVS--------EARNT-----IRSSTDELLFP--RASRDYDGGIRDEESHWHSAP
          	:    .            * :             *      .                 .

MG04836.1 	LVLALLPAIAGLLFKNGSDFVTDALLLALAAVILHAAITKPW1TWYQLAQEIRLVDQVPD
NCU00127.1	LAFALLPAIAGMFHNKGSDFITDIILIVLAAIFLHWSLTVPW2NMYKSAQQVRLENEQ--
FG07271.1 	LAFAFLPAISGLLFKNGSAVVTDFMLLGLAGVFLNWSVTQPW2TWYHSAQQVRIQHEVVA
AN7744.1  	LGLALLPAVAGIFFQNGSAFVTDVTLLALAAVFLNWSVRLPW2EWYRSARTVRRQNHLYD
          	* :*:***::*::.::** .:**  *: **.::*: ::  **   *: *: :*  ..   

MG04836.1 	VVHESSDQDDELSCEDGESMDDEYSFKPRTRARLGKKRKTS------------------P
NCU00127.1	IVESALDEDGTSPTVTAIQTPSVEVEEPRTPIESKQTTSTSDENAKLDSPEIPIAPQQEP
FG07271.1 	DSVIDDDSDLDSSTHGPGPNSPLDHVPEDEAVHAEPTEEIH-------------------
AN7744.1  	IAPPMGIDRPISPTHDSHEDGDLPDGDEEDGHHKER------------------------
          	       .    .                   .                           

MG04836.1 	GETKLTERQKQAMAELRLHETFALVSCFAFPVLGAILLHVIRSQLSRPSEGLVSDYNLAI
NCU00127.1	HSPRHTRRVDKALGELYKIESLALVLCFVSPLMMAYLLQIMRNQFKSEG-GLVSDFSLTL
FG07271.1 	-EREPSKQQQDALAELYFYEIIALASCFLLPLLGAYLLHAIRSQLSRPSEGLVSNYNLTI
AN7744.1  	--IAPQSTSAAAAEELQIHELAALASCFLFPLIGTWILHTIRSNLSRPSEGLVSNYNLTI
          	           *  **   *  **. **  *:: : :*: :*.::.  ..****::.*::

MG04836.1 	FILAAEIRPVRHLLHLFESRTWRMQRIVASNPFLK------------SEPQSIALQLDHL
NCU00127.1	YILAAEVRPFSHLIKLIQARTLHLQRIVHKNPYSESAI-------TPTKLQGILERLGDL
FG07271.1 	FLLMAEFRALSHVIKLVQSRTLHLQRVVQGGPS------------KFQQVNKNTEQLETV
AN7744.1  	FLLAAEIRPFSHLLKMVQSRTLHLQRIMASSDDDEYGNKDKIDVGKIIDLSNRLEELEAH
          	::* **.*.. *::::.::** ::**::  .   . . ... . ..  . .    .*   

MG04836.1 	SERIDQLETR-------------AAADDTDAISEATSQRREAILRDAKTQIQPEVKALYR
NCU00127.1	EQRATGLKPSNGSANNLDNNGQNDGSEGGQDQQNLNPKVRDGMVREVRNTIQPDLDALNR
FG07271.1 	LARLERLESRVTNDETSTVQDIKQEISKTKQKD-------SAVARDVRNAIQPELDALNR
AN7744.1  	VAETAALRIASTTNVEKENETKKENREPTPQEQEYTFSLVSEAAAKYHKGFQADIDALNR
          	  .   *.    .      .  .   .     .. . .  .    . :. :*.::.** *

MG04836.1 	TVRQFEKKYIVLAVQVDNRLHLLDRRIEDAITVAAHAAKS----CRPRPSLLRTTSEQ--
NCU00127.1	AVRRYEKKATVLAYQTEARLGALDGRLNDAIALAAAAAKAGGTGARPHGNNSRATGASGA
FG07271.1 	AVRRYEKKATLLQIQTESRFSGVEARLDDAIALAASAAKN--------SASHKNVFVWAM
AN7744.1  	AVRQYEKRTALTAYQTETRLQVLEAKTRDALSLAATAQRS--------TTHRS-------
          	:**::**:  :   *.: *:  :: :  **:::** * :                     

MG04836.1 	-----TLGLLFLPVQITKSLLSLPFR-LLGTAVG~LFFRAPRKTNRGYRSGRSSRSSSAC
NCU00127.1	NSGLSLFGLLAKALDTIVWAAAMPFQALLGLVVL~FPLRAVAGFLKGVTGGSSSRSRIAV
FG07271.1 	ESLTAAFLLPFRTLLRIMLLPLSTIFALMSKGKK~RNSPPVKSSSRSSRNGKTVVQPKYN
AN7744.1  	-----------------QGLGLGLFDSLLSRTIK0AWGEDM-------------------
          	                        : :*:.                              

MG04836.1 	GD~RSSRASSRR---~-------------------------------------------~
NCU00127.1	GK0SMLKHTMTDGED1ILPGCGVSRSVDVAEGRLGYIEIEVGRSGGGGGLENKARGIGR2
FG07271.1 	GD~RVPSRVAKR---~-------------------------------------------~
AN7744.1  	--~------------~-------------------------------------------~
          	                                                            

MG04836.1 	------------------------
NCU00127.1	KLFLNLEMGGTWEAGAREEGRFGD
FG07271.1 	------------------------
AN7744.1  	------------------------
          	
```
